# Supplementary material for: Cortical potentials evoked by stimulation of cervical vagus vs. auricular nerve: a comparative, parametric study in nonhuman primates
Source: bioRxiv. 2025 Dec 31:2025.12.30.697054. Preprint. [Version 1] doi: 10.64898/2025.12.30.697054 (PMC12776391; doi:10.64898/2025.12.30.697054)
Supplement: Supplement 1 [file media-1.pdf]

Supplementary Materials

Cortical potentials evoked by stimulation of cervical vagus vs. auricular nerve: A comparative, parametric study in nonhuman primates

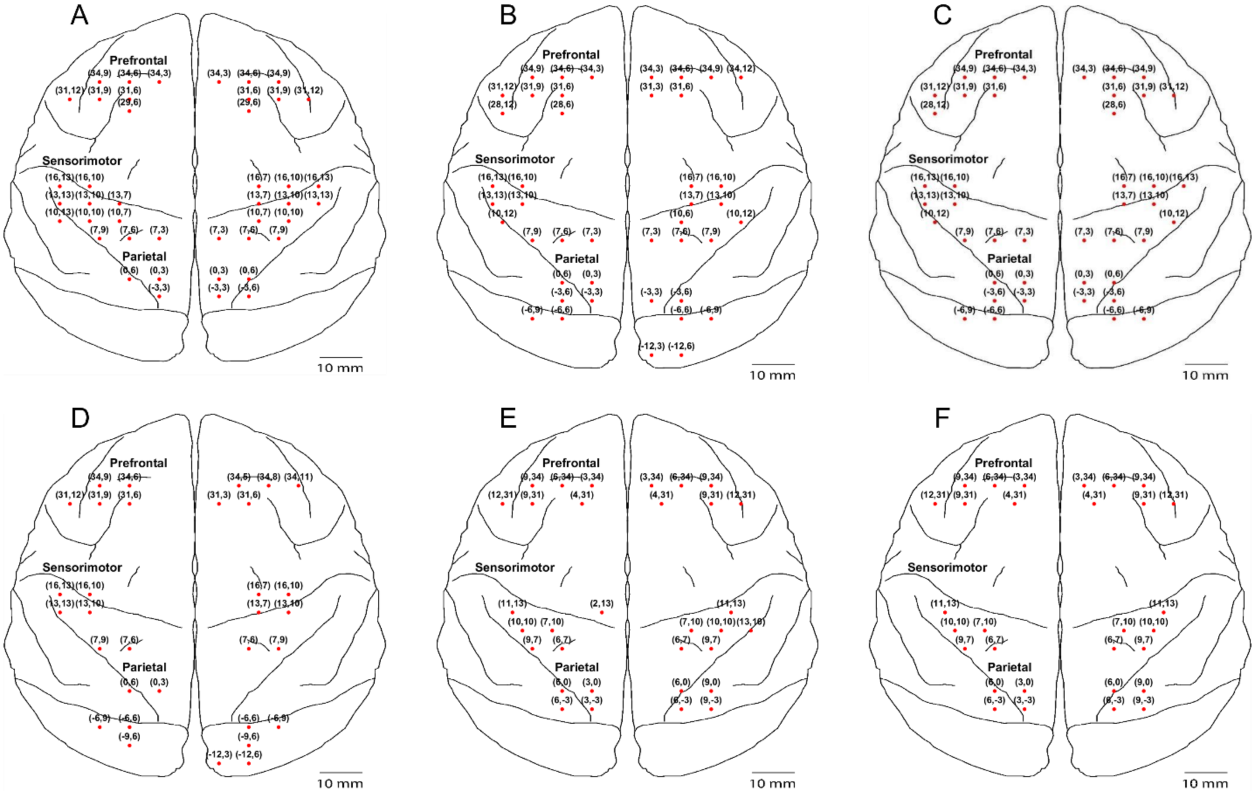

Supplementary Figure S1. Cortical locations of implanted electrodes on the cortical map for Bean (A), Hugo (B), Gomez (C), Inigo (D), Draco (E), and Zhivago (F). For each site, the numbers in parentheses indicate the stereotaxic coordinates: medial-lateral (distance from midline in mm), and anterior-posterior (distance from anterior commissure, in mm).

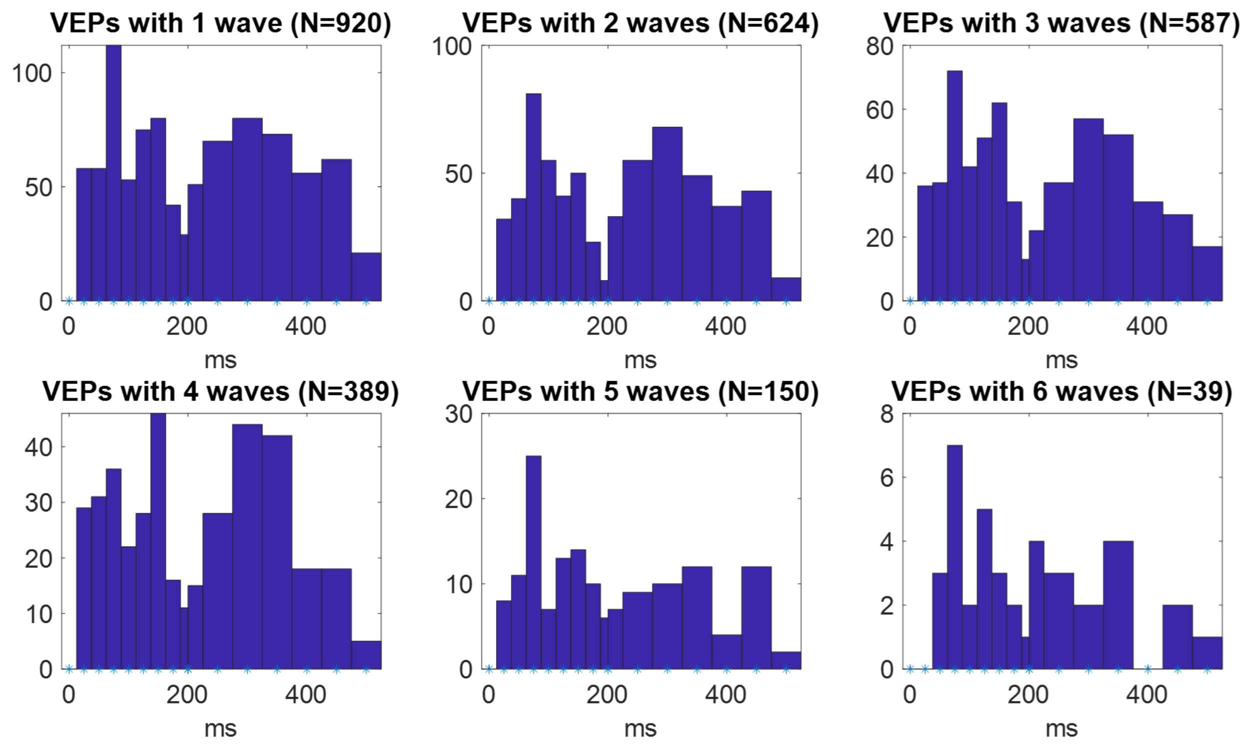

Supplementary Figure S2. Latency distributions of detected waves in subsets of VEPs with 1, 2, 3, etc waves.

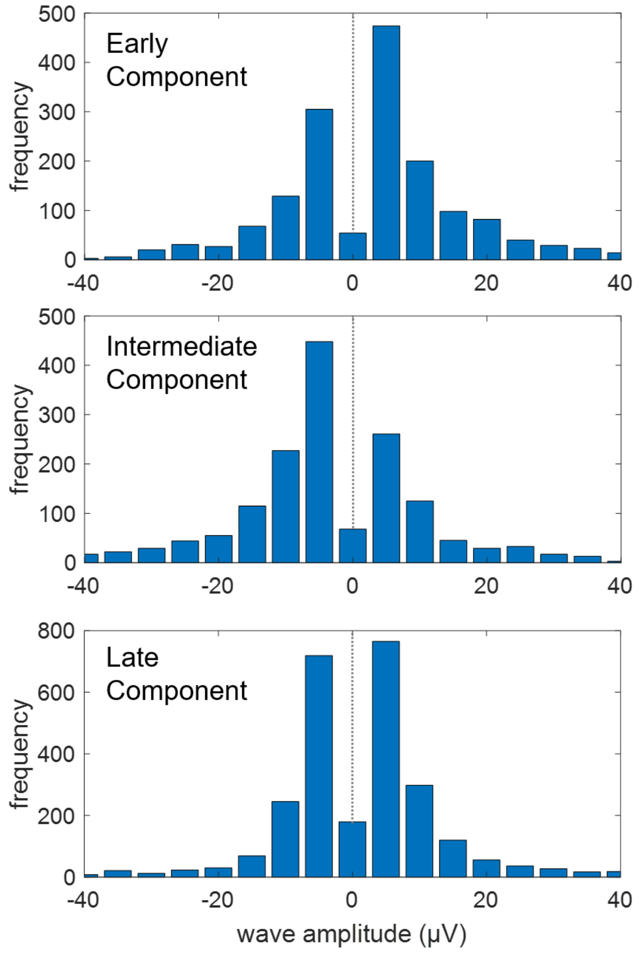

Supplementary Figure S3. Amplitude distributions of detected waves classified as early (top), intermediate (middle) or late components (bottom).

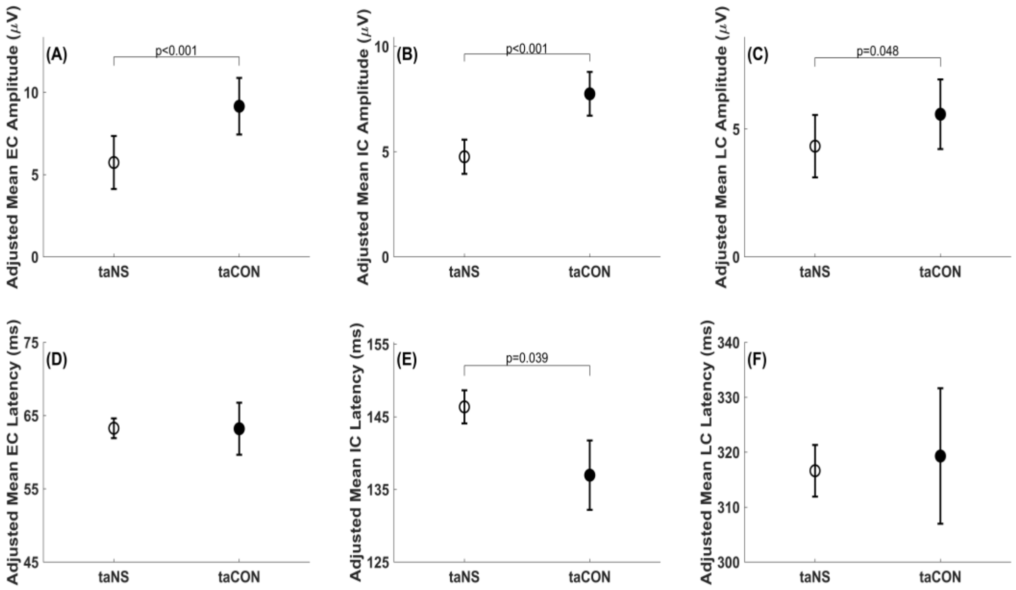

Supplementary Figure S4. Overall comparison of VEPs between taNS and trans-auricular control stimulation (taCON), applied to the earlobe. (A-C) Adjusted means for EC, IC and LC amplitudes. (D-F) Adjusted means for EC, IC and LC latencies.

Supplementary Table S1. Breakdown of the stimulation protocols used in each monkey

Monkeys					N. Pulses group	Intens-ity group	Freq-ency group	Pulse width	Stim site	N. trials
Bean	Gomez	Hugo	Inigo		5–20	1000–1499	30–100	100	taNS	67
Zhivago					5–20	≥2000	>100	200	cvns	45
Bean	Gomez	Hugo	Inigo		5–20	1000–1499	30–100	200	taNS	44
Bean	Draco	Hugo	Inigo	Zhivago	5–20	1000–1499	>100	200	taNS	41
Bean	Gomez	Hugo			5–20	≥2000	30–100	100	taNS	40
Draco	Zhivago				5–20	1000–1499	>100	200	cvns	39
Draco	Inigo				5–20	<1000	30–100	200	cvns	36
Draco	Inigo				5–20	<1000	>100	200	cvns	34
Bean	Draco	Hugo	Zhivago		5–20	≥2000	>100	200	taNS	32
Bean	Gomez	Hugo	Inigo		>20	1000–1499	>100	100	taNS	31
Bean	Draco	Gomez	Hugo		5–20	<1000	>100	200	taNS	27
Draco	Gomez	Inigo			5–20	1000–1499	30–100	200	cvns	25
Draco	Zhivago				5–20	1500–1999	>100	200	cvns	24

Inigo					5–20	<1000	30–100	100	cvns	23
Bean	Hugo	Zhivago			5–20	1500–1999	>100	200	taNS	23
Gomez					5–20	1000–1499	30–100	100	cvns	21
Bean	Gomez	Hugo			>20	≥2000	>100	100	taNS	16
Draco	Gomez	Zhivago			5–20	1500–1999	30–100	200	cvns	15
Zhivago					5–20	≥2000	30–100	200	cvns	14
Gomez	Inigo				>20	1000–1499	>100	200	taNS	14
Gomez					5–20	1500–1999	30–100	100	cvns	13
Bean	Gomez	Hugo			1–4	≥2000	0–29	200	taNS	11
Inigo					>20	<1000	>100	200	cvns	11
Gomez	Inigo				5–20	1000–1499	>100	100	cvns	10
Bean	Gomez	Hugo			5–20	≥2000	30–100	200	taNS	9
Bean	Draco	Hugo	Zhivago		5–20	≥2000	>100	200	ta Control	9
Inigo					>20	<1000	>100	100	cvns	9
Bean	Zhivago				5–20	1500–1999	>100	200	ta Control	8
Hugo	Inigo				1–4	1000–1499	0–29	100	taNS	7
Bean	Gomez	Hugo	Inigo		1–4	1000–1499	0–29	200	taNS	7
Bean	Gomez	Hugo			5–20	<1000	30–100	200	taNS	7
Bean	Inigo				5–20	1000–1499	>100	100	taNS	7
Bean					5–20	≥2000	>100	100	taNS	7
Gomez	Inigo				1–4	<1000	0–29	100	cvns	6
Gomez	Inigo				1–4	1500–1999	0–29	200	cvns	6
Bean	Hugo	Inigo			5–20	1500–1999	30–100	200	taNS	6
Bean					5–20	1500–1999	>100	100	taNS	6
Gomez					>20	1000–1499	>100	100	cvns	6
Gomez					>20	1500–1999	>100	100	cvns	6
Bean	Draco				5–20	1000–1499	>100	200	ta Control	5
Bean	Hugo	Inigo			1–4	1500–1999	0–29	200	taNS	4
Hugo					1–4	≥2000	0–29	100	taNS	4
Inigo					1–4	<1000	0–29	200	cvns	3

Gomez	Inigo				5–20	<1000	>100	100	cvns	3
Gomez					5–20	1500–1999	>100	100	cvns	3
Gomez					>20	1000–1499	>100	200	cvns	3
Gomez					1–4	1000–1499	0–29	100	cvns	2
Gomez					1–4	1500–1999	0–29	100	cvns	2
Bean					5–20	1500–1999	30–100	100	taNS	2
Bean					5–20	1500–1999	30–100	200	ta Control	2
Gomez					>20	≥2000	>100	200	taNS	2
Bean					5–20	1000–1499	30–100	200	ta Control	1
Bean					5–20	≥2000	30–100	200	ta Control	1
Gomez					5–20	≥2000	>100	100	cvns	1

Supplementary Table S2. LMM beta estimates for EC amplitude

Effect			Beta Estimate	Standard Error	Lower CL	Upper CL	Pr > |t|
**Intercept**	Variable	Interaction variable	2.20	2.17	-2.05	6.46	0.3568
**Stimulation site**	cVNS		-1.36	2.08	-5.44	2.72	0.5134
	taNS		0.00	.			.
**Brain region**	PC		-0.79	0.67	-2.09	0.51	0.2355
	PFC		-1.29	0.65	-2.56	-0.02	0.0458
	SM		0.00	.			.
**# of pulses**	Intermediate [5-20)		-0.12	0.64	-1.36	1.13	0.8544
	Long 20+		-1.77	1.11	-3.94	0.40	0.1093
	Short [1-4]		0.00	.			.
**Intensity**	2000+		3.71	1.14	1.47	5.95	0.0012
	[1000-1500)		1.88	1.10	-0.29	4.04	0.0893
	[1500-2000)		2.11	1.32	-0.48	4.70	0.1099
	<1000		0.00	.			.
**Pulse width**	100		-1.53	0.62	-2.76	-0.31	0.0142
	200		0.00	.			.
**Frequency**	High 100+ Hz		3.38	1.20	1.03	5.73	0.0048
	Medium [30-100 Hz]		0.93	1.05	-1.13	2.99	0.3756
	Low [0-30 Hz)		0.00	.			.

**Laterality**	Contralateral		0.08	0.53	-0.97	1.13	0.885
	Ipsilateral		0.00	.			.
**Stimulation site * Brain region**	cVNS	PC	5.34	0.95	3.47	7.21	<.0001
	cVNS	PFC	6.02	0.94	4.18	7.86	<.0001
	cVNS	SM	0.00	.			.
	taNS	PC	0.00	.			.
	taNS	PFC	0.00	.			.
	taNS	SM	0.00	.			.
**Stimulation site * # of pulses**	cVNS	Intermediate [5-20)	2.16	0.91	0.37	3.95	0.0183
	cVNS	Long 20+	-1.06	1.56	-4.12	2.00	0.4964
	cVNS	Short [1-4]	0.00	.			.
	taNS	Intermediate [5-20)	0.00	.			.
	taNS	Long 20+	0.00	.			.
	taNS	Short [1-4]	0.00	.			.
**Stimulation site * Intensity**	cVNS	2000+	1.26	1.82	-2.30	4.83	0.4869
	cVNS	[1000-1500)	1.95	1.38	-0.75	4.65	0.1576
	cVNS	[1500-2000)	0.62	1.58	-2.49	3.73	0.6956
	cVNS	<1000	0.00	.			.
	taNS	2000+	0.00	.			.
	taNS	[1000-1500)	0.00	.			.
	taNS	[1500-2000)	0.00	.			.
	taNS	<1000	0.00	.			.
**Stimulation site * Pulse width**	cVNS	100	-0.33	1.01	-2.32	1.66	0.7437
	cVNS	200	0.00	.			.
	taNS	100	0.00	.			.
	taNS	200	0.00	.			.
**Stimulation site * Frequency**	cVNS	High 100+ Hz	2.14	1.81	-1.41	5.68	0.2385
	cVNS	Medium [30-100 Hz]	-1.43	1.68	-4.72	1.86	0.3941
	cVNS	Low [0-30 Hz)	0.00	.			.
	taNS	High 100+ Hz	0.00	.			.
	taNS	Medium [30-100 Hz]	0.00	.			.
	taNS	Low [0-30 Hz)	0.00	.			.
**Stimulation site * Laterality**	cVNS	Contralateral	-2.55	0.77	-4.06	-1.03	0.001
	cVNS	Ipsilateral	0.00	.			.

	taNS	Contralateral	0.00	.			.
	taNS	Ipsilateral	0.00	.			.

Supplementary Table S3. LMM beta estimate for the EC latency

Effect			Beta Estimate	Standard Error	Lower CL	Upper CL	Pr > |t|
**Intercept**			51.64	4.58	42.66	60.63	<.0001
**Stimulation site**	cVNS		11.37	5.80	0.00	22.75	0.0503
	taNS		0.00	.			.
**Brain region**	PC		3.23	1.65	0.00	6.46	0.0504
	PFC		0.08	1.60	-3.06	3.22	0.9602
	SM		0.00	.			.
**# of pulses**	Intermediate [5-20)		0.99	1.59	-2.11	4.10	0.5306
	Long 20+		-5.49	2.57	-10.52	-0.47	0.0324
	Short [1-4]		0.00	.			.
**Intensity**	2000+		2.47	3.32	-4.04	8.98	0.4567
	[1000-1500)		3.49	3.34	-3.05	10.03	0.2956
	[1500-2000)		4.82	3.61	-2.26	11.89	0.1823
	<1000		0.00	.			.
**Pulse width**	100		2.01	1.66	-1.23	5.26	0.2243
	200		0.00	.			.
**Frequency**	High 100+ Hz		9.20	3.31	2.71	15.70	0.0055
	Medium [30-100 Hz]		7.52	3.20	1.26	13.79	0.0187
	Low [0-30 Hz)		0.00	.			.
**Laterality**	Contralateral		-4.00	1.34	-6.62	-1.38	0.0028
	Ipsilateral		0.00	.			.
**Stimulation site x Brain region**	cVNS	PC	0.36	2.30	-4.15	4.88	0.8744
	cVNS	PFC	2.05	2.22	-2.29	6.40	0.3549
	cVNS	SM	0.00	.			.
	taNS	PC	0.00	.			.
	taNS	PFC	0.00	.			.
	taNS	SM	0.00	.			.
**Stimulation site x # of pulses**	cVNS	Intermediate [5-20)	-2.16	2.21	-6.48	2.16	0.3277
	cVNS	Long 20+	1.33	3.50	-5.53	8.19	0.7036
	cVNS	Short [1-4]	0.00	.			.
	taNS	Intermediate [5-20)	0.00	.			.
	taNS	Long 20+	0.00	.			.
	taNS	Short [1-4]	0.00	.			.
	cVNS	2000+	-2.82	4.06	-10.78	5.14	0.4878

Stimulation site x Intensity	cVNS	[1000-1500)	-5.63	3.91	-13.28	2.03	0.1500
	cVNS	[1500-2000)	-5.94	4.17	-14.12	2.24	0.1548
	cVNS	<1000	0.00	.			.
	taNS	2000+	0.00	.			.
	taNS	[1000-1500)	0.00	.			.
	taNS	[1500-2000)	0.00	.			.
	taNS	<1000	0.00	.			.
**Stimulation site x Pulse width**	cVNS	100	-5.82	2.45	-10.63	-1.02	0.0176
	cVNS	200	0.00	.			.
	taNS	100	0.00	.			.
	taNS	200	0.00	.			.
**Stimulation site x Frequency**	cVNS	High 100+ Hz	0.05	4.83	-9.42	9.52	0.9913
	cVNS	Medium [30-100 Hz]	-3.24	4.69	-12.44	5.95	0.4892
	cVNS	Low [0-30 Hz)	0.00	.			.
	taNS	High 100+ Hz	0.00	.			.
	taNS	Medium [30-100 Hz]	0.00	.			.
	taNS	Low [0-30 Hz)	0.00	.			.
**Stimulation site x Laterality**	cVNS	Contralateral	3.71	1.84	0.11	7.31	0.0436
	cVNS	Ipsilateral	0.00	.			.
	taNS	Contralateral	0.00	.			.
	taNS	Ipsilateral	0.00	.			.

Supplementary Table S4. LMM beta estimates for IC amplitude

Effect			Beta Estimate	Standard Error	Lower CL	Upper CL	Pr > |t|
**Intercept**			0.21	1.59	-2.91	3.32	0.9023
**Stimulation site**	cVNS		2.52	1.95	-1.31	6.35	0.1972
	taNS		0.00	.			.
**Brain region**	PC		-1.35	0.63	-2.58	-0.12	0.0316
	PFC		-1.91	0.61	-3.11	-0.72	0.0017
	SM		0.00	.			.
**# of pulses**	Intermediate [5-20)		0.41	0.60	-0.76	1.59	0.4876
	Long 20+		-1.22	1.04	-3.26	0.82	0.2412
	Short [1-4]		0.00	.			.
**Intensity**	2000+		4.36	1.07	2.25	6.47	<.0001
	[1000-1500)		2.24	1.04	0.20	4.28	0.0316
	[1500-2000)		3.17	1.24	0.73	5.61	0.0108
	<1000		0.00	.			.

**Pulse width**	100		-1.51	0.59	-2.66	-0.36	0.0104
	200		0.00	.			.
**Frequency**	High 100+ Hz		4.03	1.13	1.82	6.23	0.0003
	Medium [30-100 Hz]		0.93	0.99	-1.01	2.87	0.3453
	Low [0-30 Hz)		0.00	.			.
**Laterality**	Contralateral		0.25	0.50	-0.74	1.24	0.6232
	Ipsilateral		0.00	.			.
**Stimulation site * Brain region**	cVNS	PC	2.20	0.90	0.44	3.97	0.0145
	cVNS	PFC	-0.20	0.89	-1.94	1.54	0.8196
	cVNS	SM	0.00	.			.
	taNS	PC	0.00	.			.
	taNS	PFC	0.00	.			.
	taNS	SM	0.00	.			.
**Stimulation site * # of pulses**	cVNS	Intermediate [5-20)	-0.36	0.86	-2.05	1.33	0.6786
	cVNS	Long 20+	-1.11	1.47	-3.99	1.77	0.4488
	cVNS	Short [1-4]	0.00	.			.
	taNS	Intermediate [5-20)	0.00	.			.
	taNS	Long 20+	0.00	.			.
	taNS	Short [1-4]	0.00	.			.
**Stimulation site * Intensity**	cVNS	2000+	3.62	1.67	0.35	6.89	0.0302
	cVNS	[1000-1500)	0.00	1.30	-2.54	2.54	0.9985
	cVNS	[1500-2000)	1.31	1.49	-1.61	4.24	0.3786
	cVNS	<1000	0.00	.			.
	taNS	2000+	0.00	.			.
	taNS	[1000-1500)	0.00	.			.
	taNS	[1500-2000)	0.00	.			.
	taNS	<1000	0.00	.			.
**Stimulation site * Pulse width**	cVNS	100	-0.61	0.95	-2.47	1.25	0.5184
	cVNS	200	0.00	.			.
	taNS	100	0.00	.			.
	taNS	200	0.00	.			.
**Stimulation site * Frequency**	cVNS	High 100+ Hz	2.95	1.71	-0.39	6.30	0.0836
	cVNS	Medium [30-100 Hz]	-0.50	1.58	-3.60	2.61	0.7542
	cVNS	Low [0-30 Hz)	0.00	.			.
	taNS	High 100+ Hz	0.00	.			.
	taNS	Medium [30-100 Hz]	0.00	.			.
	taNS	Low [0-30 Hz)	0.00	.			.
	cVNS	Contralateral	-0.94	0.73	-2.36	0.49	0.1967

**Stimulation site * Laterality**	cVNS	Ipsilateral	0.00	.			.
	taNS	Contralateral	0.00	.			.
	taNS	Ipsilateral	0.00	.			.

Supplementary Table S5. LMM beta estimates for IC latency

Effect			Beta Estimate	Standard Error	Lower CL	Upper CL	Pr > |t|
**Intercept**			139.99	6.44	127.38	152.60	<.0001
**Stimulation site**	cVNS		-4.98	7.55	-19.78	9.83	0.5102
	taNS		0.00	.			.
**Brain region**	PC		3.97	2.31	-0.56	8.49	0.0859
	PFC		9.37	2.17	5.12	13.62	<.0001
	SM		0.00	.			.
**# of pulses**	Intermediate [5-20)		3.26	2.20	-1.05	7.57	0.1386
	Long 20+		3.95	3.46	-2.82	10.73	0.2529
	Short [1-4]		0.00	.			.
**Intensity**	2000+		-3.10	4.43	-11.79	5.58	0.4838
	[1000-1500)		1.92	4.47	-6.84	10.67	0.6682
	[1500-2000)		-7.64	4.82	-17.08	1.80	0.1131
	<1000		0.00	.			.
**Pulse width**	100		0.86	2.24	-3.54	5.25	0.7016
	200		0.00	.			.
**Frequency**	High 100+ Hz		0.43	4.42	-8.24	9.10	0.9224
	Medium [30-100 Hz]		5.78	4.26	-2.57	14.12	0.1752
	Low [0-30 Hz)		0.00	.			.
**Laterality**	Contralateral		1.78	1.85	-1.84	5.40	0.3363
	Ipsilateral		0.00	.			.
**Stimulation site * Brain region**	cVNS	PC	1.40	3.15	-4.77	7.57	0.6558
	cVNS	PFC	-4.50	3.05	-10.49	1.49	0.1408
	cVNS	SM	0.00	.			.
	taNS	PC	0.00	.			.
	taNS	PFC	0.00	.			.
	taNS	SM	0.00	.			.
**Stimulation site * # of pulses**	cVNS	Intermediate [5-20)	0.20	3.05	-5.77	6.18	0.9467
	cVNS	Long 20+	10.08	4.65	0.97	19.20	0.0303
	cVNS	Short [1-4]	0.00	.			.
	taNS	Intermediate [5-20)	0.00	.			.
	taNS	Long 20+	0.00	.			.

	taNS	Short [1-4]	0.00	.			.
**Stimulation site * Intensity**	cVNS	2000+	3.15	5.88	-8.36	14.67	0.5914
	cVNS	[1000-1500)	-3.24	5.40	-13.82	7.34	0.5481
	cVNS	[1500-2000)	7.61	5.72	-3.61	18.83	0.1838
	cVNS	<1000	0.00	.			.
	taNS	2000+	0.00	.			.
	taNS	[1000-1500)	0.00	.			.
	taNS	[1500-2000)	0.00	.			.
	taNS	<1000	0.00	.			.
**Stimulation site * Pulse width**	cVNS	100	2.73	3.31	-3.75	9.22	0.409
	cVNS	200	0.00	.			.
	taNS	100	0.00	.			.
	taNS	200	0.00	.			.
**Stimulation site * Frequency**	cVNS	High 100+ Hz	-4.48	6.13	-16.51	7.54	0.4648
	cVNS	Medium [30-100 Hz]	1.18	5.89	-10.36	12.72	0.8409
	cVNS	Low [0-30 Hz)	0.00	.			.
	taNS	High 100+ Hz	0.00	.			.
	taNS	Medium [30-100 Hz]	0.00	.			.
	taNS	Low [0-30 Hz)	0.00	.			.
**Stimulation site * Laterality**	cVNS	Contralateral	1.28	2.55	-3.71	6.28	0.6147
	cVNS	Ipsilateral	0.00	.			.
	taNS	Contralateral	0.00	.			.
	taNS	Ipsilateral	0.00	.			.

Supplementary Table S6. LMM beta estimates for LC amplitude

Effect			Beta Estimate	Standard Error	Lower CL	Upper CL	Pr > |t|
**Intercept**			1.30	1.85	-2.32	4.93	0.5124
**Stimulation site**	cVNS		3.97	1.78	0.48	7.45	0.0256
	taNS		0.00	.			.
**Brain region**	PC		-0.48	0.57	-1.60	0.63	0.3971
	PFC		-1.38	0.55	-2.46	-0.29	0.0127
	SM		0.00	.			.
**# of pulses**	Intermediate [5-20)		0.18	0.54	-0.88	1.24	0.741
	Long 20+		0.77	0.95	-1.09	2.62	0.4178
	Short [1-4]		0.00	.			.
**Intensity**	2000+		2.74	0.98	0.83	4.65	0.005
	[1000-1500)		2.20	0.94	0.35	4.05	0.02

	[1500-2000)		2.89	1.13	0.68	5.11	0.0104
	<1000		0.00	.			.
**Pulse width**	100		-1.08	0.53	-2.13	-0.04	0.0421
	200		0.00	.			.
**Frequency**	High 100+ Hz		2.04	1.02	0.04	4.05	0.0459
	Medium [30-100 Hz]		1.50	0.90	-0.26	3.26	0.0947
	Low [0-30 Hz)		0.00	.			.
**Laterality**	Contralateral		0.05	0.46	-0.84	0.95	0.9084
	Ipsilateral		0.00	.			.
**Stimulation site x Brain region**	cVNS	PC	2.51	0.82	0.91	4.11	0.0021
	cVNS	PFC	4.17	0.80	2.59	5.74	<.0001
	cVNS	SM	0.00	.			.
	taNS	PC	0.00	.			.
	taNS	PFC	0.00	.			.
	taNS	SM	0.00	.			.
**Stimulation site x # of pulses**	cVNS	Intermediate [5-20)	1.04	0.78	-0.49	2.57	0.1843
	cVNS	Long 20+	-1.04	1.33	-3.65	1.57	0.4342
	cVNS	Short [1-4]	0.00	.			.
	taNS	Intermediate [5-20)	0.00	.			.
	taNS	Long 20+	0.00	.			.
	taNS	Short [1-4]	0.00	.			.
**Stimulation site x Intensity**	cVNS	2000+	-5.68	1.55	-8.72	-2.64	0.0003
	cVNS	[1000-1500)	-4.94	1.18	-7.25	-2.64	<.0001
	cVNS	[1500-2000)	-6.89	1.35	-9.54	-4.23	<.0001
	cVNS	<1000	0.00	.			.
	taNS	2000+	0.00	.			.
	taNS	[1000-1500)	0.00	.			.
	taNS	[1500-2000)	0.00	.			.
	taNS	<1000	0.00	.			.
**Stimulation site x Pulse width**	cVNS	100	-0.25	0.87	-1.95	1.45	0.7724
	cVNS	200	0.00	.			.
	taNS	100	0.00	.			.
	taNS	200	0.00	.			.
**Stimulation site x Frequency**	cVNS	High 100+ Hz	1.23	1.55	-1.80	4.26	0.4256
	cVNS	Medium [30-100 Hz]	-1.37	1.44	-4.18	1.45	0.3417
	cVNS	Low [0-30 Hz)	0.00	.			.
	taNS	High 100+ Hz	0.00	.			.
	taNS	Medium [30-100 Hz]	0.00	.			.

	taNS	Low [0-30 Hz)	0.00	.			.
**Stimulation site x Laterality**	cVNS	Contralateral	0.71	0.66	-0.58	2.00	0.282
	cVNS	Ipsilateral	0.00	.			.
	taNS	Contralateral	0.00	.			.
	taNS	Ipsilateral	0.00	.			.

Supplementary Table S7. LMM beta estimates for LC latency

Effect			Beta Estimate	Standard Error	Lower CL	Upper CL	Pr > |t|
**Intercept**			318.93	17.49	284.65	353.21	<.0001
**Stimulation site**	cVNS		25.00	21.86	-17.84	67.84	0.2528
	taNS		0.00	.			.
**Brain region**	PC		-7.17	6.64	-20.19	5.85	0.2806
	PFC		-0.78	6.30	-13.12	11.57	0.9017
	SM		0.00	.			.
**# of pulses**	Intermediate [5-20)		-6.77	6.28	-19.07	5.54	0.2814
	Long 20+		-4.21	10.25	-24.31	15.88	0.6811
	Short [1-4]		0.00	.			.
**Intensity**	2000+		-24.51	12.37	-48.75	-0.27	0.0477
	[1000-1500)		-6.85	12.30	-30.95	17.26	0.5779
	[1500-2000)		-23.39	13.75	-50.33	3.56	0.0891
	<1000		0.00	.			.
**Pulse width**	100		28.80	6.31	16.42	41.17	<.0001
	200		0.00	.			.
**Frequency**	High 100+ Hz		17.32	13.44	-9.03	43.67	0.1979
	Medium [30-100 Hz]		21.70	12.64	-3.08	46.47	0.0862
	Low [0-30 Hz)		0.00	.			.
**Laterality**	Contralateral		-11.40	5.30	-21.79	-1.01	0.0316
	Ipsilateral		0.00	.			.
**Stimulation site x Brain region**	cVNS	PC	7.80	9.21	-10.26	25.85	0.3977
	cVNS	PFC	0.45	8.80	-16.80	17.70	0.9592
	cVNS	SM	0.00	.			.
	taNS	PC	0.00	.			.
	taNS	PFC	0.00	.			.
	taNS	SM	0.00	.			.
**Stimulation site x # of pulses**	cVNS	Intermediate [5-20)	18.73	8.69	1.70	35.76	0.0313
	cVNS	Long 20+	18.02	13.70	-8.84	44.88	0.1886
	cVNS	Short [1-4]	0.00	.			.

	taNS	Intermediate [5-20)	0.00	.			.
	taNS	Long 20+	0.00	.			.
	taNS	Short [1-4]	0.00	.			.
**Stimulation site x Intensity**	cVNS	2000+	-19.40	15.38	-49.54	10.75	0.2074
	cVNS	[1000-1500)	3.19	14.45	-25.12	31.51	0.8251
	cVNS	[1500-2000)	-10.64	15.85	-41.72	20.43	0.5022
	cVNS	<1000	0.00	.			.
	taNS	2000+	0.00	.			.
	taNS	[1000-1500)	0.00	.			.
	taNS	[1500-2000)	0.00	.			.
	taNS	<1000	0.00	.			.
**Stimulation site x Pulse width**	cVNS	100	-21.59	9.17	-39.56	-3.63	0.0186
	cVNS	200	0.00	.			.
	taNS	100	0.00	.			.
	taNS	200	0.00	.			.
**Stimulation site x Frequency**	cVNS	High 100+ Hz	-61.13	18.50	-97.39	-24.88	0.001
	cVNS	Medium [30-100 Hz]	-52.02	17.59	-86.49	-17.55	0.0031
	cVNS	Low [0-30 Hz)	0.00	.			.
	taNS	High 100+ Hz	0.00	.			.
	taNS	Medium [30-100 Hz]	0.00	.			.
	taNS	Low [0-30 Hz)	0.00	.			.
**Stimulation site x Laterality**	cVNS	Contralateral	22.31	7.34	7.93	36.69	0.0024
	cVNS	Ipsilateral	0.00	.			.
	taNS	Contralateral	0.00	.			.
	taNS	Ipsilateral	0.00	.			.

Supplementary Table S8. Output of the LMM for ta-control vs. taNS, for amplitude and latency of the early component (EC), intermediate component (IC) and late component (LC)

Outcome	Factor Level	LS-Mean*	95% Confidence Interval		p-value	ICC
EC, Latency	taCON	63.21	56.24	70.18	0.9839	0.0141
	taNS	63.28	60.64	65.92		
IC, Latency	taCON	136.97	127.59	146.34	0.0388	0.0315
	taNS	146.37	141.89	150.84		
LC, Latency	taCON	319.31	295.11	343.52	0.8303	0.0073
	taNS	316.63	307.40	325.86		
EC, Amplitude	taCONT	9.16	5.77	12.54	<0.0001	0.3846
	taNS	5.74	2.58	8.90		
IC, Amplitude	taCONT	7.75	5.72	9.79	<0.0001	0.1185

	taNS	4.76	3.18	6.35		
LC, Amplitude	taCONT	5.58	2.92	8.24	0.0475	0.2800
	taNS	4.33	1.93	6.72		

** Least square means estimates were adjusted for the random effect of monkey and fixed effects of stimulation modality, brain region, number of pulses, intensity, frequency, and laterality, along with two-way interactions between stimulation site and each fixed effect.*
